# Severity in the genomic age: the significance of lived experience to understandings of severity

**DOI:** 10.1038/s41431-024-01652-5

**Published:** 2024-06-26

**Authors:** Amarpreet Kaur

**Affiliations:** University of Birmingham, Birmingham, UK

**Keywords:** Genomics, Medical genetics

## Abstract

This article explores the significance of lived experience to understandings of severity in the genomic age. It draws upon data from structured interviews with 21 people living with monogenic conditions in England. The article argues that while lived experiences are subjective, participants consider the severity of disease by the impact a condition has on a person’s quality of life and mental health; both of these interplays are influenced by social, economic, and environmental factors. The three factors and considerations to the impact of living with disease on mental health are generally absent from current frameworks designed to assess severity for clinical applications of genomic technologies such as preimplantation genetic testing (PGT). This article describes ways in which such factors impact the quality of life and the mental health of people living with genetic conditions. It also indicates what lived experiences, which illustrate the impact of these factors, have to offer policy-makers when they are assessing the concept of severity or seriousness of genetic conditions for applications of existing and potential genomic technologies in the genomic age.

## Introduction

The concept of ’severity' or ’seriousness' in this genomic age is a critical topic for several reasons. First, as genomic science and technologies continue to develop, so do the possibilities for applications in genomic medicine [1, 2]. However, many proposed applications, particularly those relating to genetic testing and heritable human genome editing (HHGE), hinge on determining which applications could be acceptable and under what conditions [3]. One such condition is often the ’severity' or ’seriousness' of a genetic disease [3]. Thus, these concepts often feature in healthcare policy-making across the globe and guide practices in genomic medicine [3–5].

Second, as will be discussed in this article, examples of people living with genetic conditions indicate that these concepts are very subjective [4–7]. To date, this subjectivity has meant that there is no agreed, global, definition of what is ’serious' or ’severe' in the context of disease [8]. This subjectivity proves to be problematic for professionals, researchers, ethicists, policy-makers, and other stakeholders involved in trying to determine potential clinical applications of genomic technologies [8, 9]. For example, the report from the International Commission on the Clinical Use of HHGE recommended that any initial applications of HHGE for reproductive purposes (in conjunction with other recommendations) be limited to serious monogenic diseases. In the report, serious monogenic diseases are defined as those which cause severe morbidity or premature death [3]. By switching between terms, from serious to severe, the report circumvents detailing any insight to what such diseases may be, and therefore any concrete direction for potential applications.

And third, further to the above issues, current frameworks to assess the ‘seriousness’ of a condition, are often limited to the biomedical aspects of a disease [4, 5, 10, 11]; this is problematic because this approach can ignore lived experiences with disease, disability, and human values [5]. Lived experiences extend beyond biomedical factors to interplay with social, environmental, and economic factors [10, 12]. An example of such a framework is the PGD decision tree used by the Human Fertilisation and Embryology Authority’s (HFEA) Statutory Approvals Committee when considering whether to licence a genetic condition for prevention by preimplantation genetic testing (PGT). This tool assesses the seriousness of a condition by taking into account the age at which it onsets, its possible symptoms and whether the condition could be fatal, whether the condition is treatable, and, if so, what type of treatment is available, the extent to which it alleviates symptoms, and whether the treatment is invasive. It also assesses the effect of the condition on the quality of life in terms of degeneration and impairments, and the variability of symptoms. The HFEA’s committee bases decisions regarding seriousness on the worst possible symptoms. While the assessment is extensive, none of those factors account the social, environmental, and economic factors which interplay with lived experiences.

This article focusses on the significance of lived experiences with genetic disease to understandings of severity, and what the relevance of these experiences could contribute to the long-standing, ongoing, discussion of ‘severity’ or ‘seriousness’ of disease in relation to healthcare policy-making in this genomic era. This article offers insights into the significance of considering social, environmental, and economic factors in deliberations relating to quality of life, and explores what the inclusion of such factors in future frameworks could mean for potential applications of HHGE. Discussions in this article stem from mixed-methods research on HHGE as a potential reproductive choice for the prevention of monogenic disease. The use of mixed methods enabled a detailed exploration of participants’ perceptions; is advantageous when analysing such a subjective and complex matter. The data is positioned in the context of broader literature and contexts to highlight their significance to understandings of severity in this genomic age.

## Participants and methods

The data presented in this article were collected as part of a larger, sequential, three-phase, mixed-methods research project which aimed to explore factors which influence attitudes towards the use of HHGE as a potential reproductive choice in the United Kingdom, and how identifying those factors could assist in the design of future regulation. Detailed information on the methods can be found in [13]. The data presented in this article focusses on analyses from the third phase of the research.

Following ethical approval from the University of Cambridge’s Department of Sociology’s Research Ethics Committee, participants were recruited online via support groups on Facebook. Expressions of interest to participate in the research were submitted via a link to an online form detailed in posts on Facebook. Participants were selected based on their provision of informed consent, which included potential uses of their data, and being affected by a monogenic condition licensed for prevention by PGT, i.e. diseases deemed ‘clinically serious’ enough for prevention with the assistance of genomic technologies. Such conditions are listed on the HFEA’s website. The conditions were also matched to one of twelve categories of disease used by the International Classification of Disease 11th Revision. The matching was exercised so that the participant sample would include a diverse range of genetic conditions. The sample’s diversity in conditions was considered to be key to exploring whether experiences of living with different types of conditions influenced participants’ views on the concept of severity.

The types of disease and the corresponding conditions covered in the participant sample are listed in Table 1. Some conditions span multiple classifications; thus, the table also illustrates these crossovers. Table 2 summarises the conditions in Table 1.

**Table 1:** List of monogenic conditions in participant sample.

Classification (Diseases of…)	Condition(s) (licensed for PGT)
Blood or blood-forming organs	Beta Thalassaemia
Circulatory system	Cardiomyopathy
Digestive system	Hirschsprung’s Disease
Ear or mastoid process	Pendred Syndrome and Stickler Syndrome
Endocrine/metabolic	Kallmann Syndrome and Schwachman–Diamond Syndrome
Immune system	Primary Immunodeficiency Disease (PID)
Musculoskeletal system	Muscular Dystrophy and Schwachman–Diamond Syndrome
Neoplasms	Breast Ovarian Cancer (BRCA2)
Nervous system	Charcot Marie Tooth Disease and Parkinson’s Disease
Neurodevelopmental disorders	Huntington’s Disease and Spina Bifida
Respiratory system	Cystic Fibrosis and Primary Ciliary Dyskinesia (PCD)
Visual system	Aniridia and Stickler Syndrome

**Table 2:** Summary of conditions in participant sample.

Condition	Summary
Aniridia	Partial or complete absence of the iris; causes reduction in visual acuity and increased sensitivity to light (photophobia).
Beta Thalassaemia	Reduced production of beta globin, resulting in a reduced capacity for blood to carry oxygen throughout the body.
Breast & Ovarian Cancer (BRCA2)	Increased risk of developing breast and/or ovarian cancers leading to greater prophylactic measures.
Cardiomyopathy	Stretching, thickening, or stiffening of the heart chambers which impedes the functioning of the heart muscle.
Charcot Marie Tooth Disease	Nerve damage, mostly to peripheral nerves, causing motor and sensory neuropathy, leading to muscle weakness and numbness.
Cystic Fibrosis	The production of abnormally thick mucus in the lungs and digestive system which causes infections and problems digesting food.
Hirschsprung’s Disease	Missing nerve cells in the colon which causes severe constipation and infections.
Huntington’s Disease	Progressive breakdown of nerve cells in the brain, causing parts of the brain to stop working properly.
Kallmann Syndrome	Underdevelopment of specific neurons which causes the delay or prevention of puberty, and an impaired sense of smell.
Muscular dystrophy	Progressive weakening and wasting of muscles, causing muscles to weaken and increased disability.
Parkinson’s Disease	Progressive degeneration of the central nervous system, causing involuntary tremors and stiffening of muscles.
Pendred Syndrome	Lack of the pendrin protein, causing early hearing loss and an enlarged thyroid gland.
Primary Ciliary Dyskinesia (PCD)	Impaired functioning of the cilia, leading to mucus build up and thus inflammation and infection in the airways, sinuses, and ears.
Primary Immunodeficiency Disease (PID)	Weakening of the immune system, allowing infections and other health problems to occur more easily.
Schwachman–Diamond Syndrome	A malfunction in bone marrow, which causes problems with the pancreas, infections, anaemia, and abnormal bleeding.
Spina Bifida	A neural tube defect in which the spinal cord or spinal bones do not form properly, potentially causing paralysis and/or incontinence.
Stickler Syndrome	A connective tissue disorder that affects the production of collagen causing ear problems, hearing loss, and problems with joints.

The in-depth, structured interviews were conducted between 30 April 2019 and 2 October 2019, and consisted of open-ended questions, polls, and a ranking activity. The questions and activities associated to the data in this article are detailed in Appendix 1. The ranking activity entailed ordering symptoms and associations with disease from most to least impactful. The symptoms/associations were derived from literature on living with disease and/or disability [12, 14–17].

All the interviews were either audio or video recorded with consent from participants. Recordings were transcribed by the author, and the transcriptions were imported to NVivo 11 for reflexive thematic analysis (RTA) [18]. During the RTA process, first codes were identified via open coding, and later themes were generated by reflecting on patterns of shared meanings between individual codes. Three of the themes pertinent to this article are, 1. Severity of disease, 2. Perceptions of symptoms of associations with disease, and 3. Interplay between symptoms of and associations with disease.

Critical reflections on the data, enabled the author, as the sole researcher on the project, to consider whether the data could be interpreted in different ways; where differing interpretations could exist, these are shared with the findings alongside the researcher’s insights (from the broader data and interactions with the participants, and participants’ respective contexts). During transcription, participants’ names and any other identifiers were replaced with pseudonyms. The pseudonyms are used to protect participants’ anonymity.

Data from the polls were quantitative and the data from the ranking exercise were quantified prior to being coded. Data from the ranking activity were imported to Microsoft Excel for quantitative analysis. The symptoms/associations were assigned ranking scores from 1–9; 9 being the total number of them. The ranking scores were then used to calculate the overall rank for each symptom/association. Participants were asked to share the reasoning for their rankings, this corresponding qualitative data was coded and interpreted in conjunction with the overall rankings. Findings from these analyses are shared in the next section.

## Results

21 adults, aged 20–58 years, 6 of whom self-identified as male and 15 as female, were selected to participate in the research. 17 participants have a monogenic condition, and of these 5 participants also have or had family members affected by the same condition. 4 participants were mothers of children with severe disease and of these, 1 participant is a carrier of their child’s disease. Participants who were asymptomatic for a genetic condition were included in the research as advocates for those with conditions who were unable to represent themselves.

### Severity of disease

Participants felt that the severity of a disease should be taken into account when considering potential applications for HHGE. This is because participants felt that only severe diseases should be prevented via potential clinical applications of genomic technologies. An example of this stance is evident in the following quote:

*I think the severity of a disease should be taken into account* [when considering what diseases could be permitted for potential applications of HHGE] *because I think there are many many conditions where people may have a disability, but they do not feel it adversely impacts the quality of their life*.

*(Resina, 47, has Beta Thalassaemia)*

This quote from Resina also reiterates that the concept of severity is entwined with the concept of quality of life and that there are some conditions which may not seem severe to those who live with them. The symptoms or associations with disease that participants were asked to rank are those which are commonly linked to impeding people’s quality of life [12, 14–17].

### Perceptions of symptoms of or associations with disease

The overall rankings of common symptoms of or associations with disease are listed in Table 3 from most to least impactful.

**Table 3:** Overall rankings of the impact of symptoms or associations with disease.

Symptom/association	Total	Rank
Pain	91	1
Dependency	86	2
No/poor treatment	83	3
Physical impairment	77	4
Cognitive impairment	76	5
Physical degeneration	74	6
Cognitive degeneration	70	7
Physiological degeneration	69	8
Physiological impairments	66	9

Table 3 indicates that, overall, participants felt that living with pain is most impactful on people’s lives and that living with physiological impairments is the least impactful. However, the supporting explanations to why these rankings were chosen, reinforce the value of considering participants’ experiences and the subjectivities that present with them according to the nature of their own condition. Participants’ explanations also revealed other experiences that they were not asked to rank but that are nonetheless impactful to or on their lives. This article focusses on the ranking of pain as an example of the significance of lived experiences. Respective reasonings for the other rankings are discussed in a separate publication (see ref. 13).

The following comments highlight the significance of lived experience with pain to participants’ ranking of it:

*Pain is high, because pain leads to everything else getting worse, because the stress with pain is just awful. And then carers, and parents, and loved ones watching pain, and there’s nothing you can do is just awful. And that’s not even being the person suffering the pain, not being able to take away pain. And with the current situation where painkillers are not prescribed very easily with people being very critical about patients in pain, and the assumption that people are taking painkillers because they’re addicted rather than because they need it, is just horrendous. Pain is just awful. And somebody suffering pain and not getting the support they need is just awful*.

(Miranda, 52, mother of child with Pendred Syndrome)

Miranda mentions that pain is ‘awful’ several times and that pain involves ‘suffering’. Her quote offers a covert account of some of the ways in which pain can impact a person’s mental health. The following quote also reflects the participant’s own experiences:

*Living in constant pain can have psychological effects on you and constant pain would be one that is best addressed. Nobody should live in pain all the time*.

(Paul, 55, has Parkinson’s Disease)

Paul highlights that pain can be ‘constant’ and overtly states that it can have ‘psychological effects’. These first two quotes indicate that the management of pain, and the pervasiveness of it on everyday life in terms of suffering and impact, can be significant to some people. However, the next quote offers a different perspective; again, because of the participant’s own lived experiences:

*I deal with a lot of pain, but I feel like it’s very low on the list. Like, if they were to stop the progression of my condition, but leave me with the pain, I’d be okay with that*.

(George, 34, has Charcot Marie Tooth Disease)

George shared that he deals with pain in addition to degeneration. The progressive nature of the disease he lives with overshadows the pain that he experiences. This does not mean that the symptom of pain is insignificant to him, but that his other symptoms are more impactful on his life. This argument is reiterated further in another quote from George:

*I am slowly losing the ability to walk, I am losing the ability to use my hands, and what I’m dealing with is getting worse and causing pain. The physical side is only one half of what I’m having to deal with, the mental baggage of having a genetic condition that progresses year-on-year is overwhelming, so overwhelming. It really affects my mental health, knowing that there’s no cure currently for a condition such as mine, is really tough*.

In this context, while physical pain may be the most impactful symptom of living with a given disease to some people, as the quote from George indicates, to others this may not. Other experiences, such as degeneration, knowing there is no cure, or the ‘mental baggage’ of living with a genetic condition could be more impactful on a person’s quality of life and therefore their perception of severity. This finding indicates that valuable insights can be gained from people’s lived experiences and the subjectivities that arise from them in terms of how symptoms of disease are perceived in relation to severity.

### Interplay between symptoms of and associations with disease

Further, although the following quote is an isolated example, several participants shared that trying to rank symptoms or associations of disease was difficult because they are often not experienced in isolation; this could also be a reason why the totals for the individual rankings are so close. Symptoms and associations tend to interplay. This finding is significant to understandings of severity in this genomic age:

*They all affect you, like, when I lose my independence, that affects me horrendously psychologically. But the loss of independence comes from all these things getting worse, so I think in a way there isn’t an order for them*. […] *But psychological health first because for me that is huge, because you have to be psychologically healthy to be able to deal with any of it, and any of these have a psychological impact on your psychological health. If you are psychologically strong, you can deal with these things better, but you can’t deal with any of it if you’re not*.

(Vivien, 48, has Cystic Fibrosis)

Understanding that lived experiences with genetic disease often encompass a range of symptoms and associations in tandem can assist comprehension of the impact that these can have on people’s lives. In isolation, any singular symptom of disease, such as pain, may not seem serious. In isolation, some experiences may seem manageable or be perceived to have little impact on a person’s quality of life. But, there are few genetic conditions, if any, where a person only has to consider one symptom or association which stems from living with it.

Additionally, although the above quotes are from a selection of participants, the impact of living with a genetic condition on participants’ mental health was expressed by them all. The psychological impacts of living with a genetic condition could therefore be significant to understandings of severity or seriousness in relation to quality of life in this genomic age.

## Discussion

The findings presented in this article offer a richness of insight that could otherwise be overlooked. However, they are indicative substances for further research and are not conclusive. The findings reinforce that understandings of severity are entwined with quality of life [8, 9]. They also suggest that while lived experiences with genetic disease are subjective, participants’ understandings of severity all included the impact of a condition on their mental health because this reportedly impacts their quality of life. Current frameworks to assess the severity of a genomic disease tend to focus on objective, biomedical factors of the condition [5] and quality adjusted life years (QALY) [8, 9]. Such frameworks rarely consider how living with a genetic condition interplays with mental health, or social, economic, and environmental factors [10, 12].

Drawing back on the findings presented in this article, this discussion will now explicitly illustrate why considerations to the three factors could be pivotal for future frameworks designed to assess potential applications of genetic technologies (whether preventative such as PGT and HHGE or as treatments via ‘gene therapy’). Miranda highlighted two issues which align with these factors. First, that obtaining prescriptions for pain medication (in England) can be difficult. Second, there is an assumption people want painkillers to feed an addiction, rather than because they need to alleviate their pain. Arguably, pain is a treatable symptom of many diseases. There are a range of effective ways to treat pain, but in some cases, depending on the reason or cause of pain, sometimes pain can be difficult to alleviate [16, 17]. Painkillers are only one method of treatment.

Some effective painkillers are only available via prescription. In England, the price of prescriptions is capped [19], and can therefore be expensive to England’s National Health Service (NHS). This can mean that some painkillers are reluctantly prescribed, or alternative medications are offered, even when specific medications could enhance a person’s quality of life [20–22], most likely based on cost-benefit analysis and QALY assessments; these are some of the reasons for the difficulties Miranda referred to. In other scenarios, alternative medicine, such as deep tissue massage, acupuncture, or chiropractic may be more effective treatments for some pains, but these are rarely offered via England’s NHS due to the expense [23], and would likely be even more difficult to access if they were. For people who cannot access or afford adequate pain relief to alleviate their pain, their quality of life is likely to be reduced by such economic factors in comparison to those who can. Further, to live with or manage pain, a person may require extra support, whether this is through physical human care and support, aids, or technology [16, 17, 24]. All such support also comes with expenses, expenses which some people may not be able to afford easily or at all. This financial burden can also impact a person’s lived experience with disease [16], and is another example of the economic impact on a person’s quality of life, and potentially their mental health.

Continuing with the example of pain for illustrative purposes relevant to the remaining two factors - living in pain can cause fatigue, irritability, and sleep deprivation. Such consequences can impact a person’s ability to concentrate, complete their daily activities, work, and socialise [16, 17, 24–28]. Hence, people living with pain can become isolated and depressed [26, 29]. These consequential effects can have profound negative impacts on the social aspects of people’s lives in addition to their mental health [25–27]. Thus, this argument sustains that the potential effects of living with a genetic disease on a person’s social life is also worthy of consideration in future frameworks.

Finally, most environments are generally designed for healthy, able-bodied people [30–32]. This can mean that people living with genetic conditions that affect their ability to conform to such environments are more likely to struggle in them or require adaptations to navigate them [12, 29]. Trying to navigate such environments can often add to people’s pains both physical and psychological, particularly if they cannot access or afford support which may make navigating ableist environments easier. This is another aspect of the reality of some people living with genetic disease face, and therefore a reasonable factor to also consider in future frameworks.

The three factors were discussed in relation to pain. However, similar arguments could be true for any of the symptoms or associations listed in the ranking exercise; this could mean that regardless of which symptoms people living with genetic conditions experience and perceive to be most severe, their quality of life and therefore their mental health are likely to be impacted by one or more of the three factors. Further, drawing back on the understanding that living with genetic disease often encompasses experiencing a range of associations and symptoms in tandem, the magnitude of such experiences on people’s quality of life and mental health when the cumulative toll of them is accounted can, in many cases, be considerable. Thus, the inclusion of the three factors and mental health in relation to quality of life in future frameworks designed to assess the severity or seriousness of a genetic disease for clinical applications of, both existing and potential, genetic technologies could offer a more holistic assessment of it.

Current frameworks to assess severe or serious diseases for clinical applications are problematic because they do not enable a holistic assessment of the impact of a given disease on people’s quality of life, in the contexts they live. Further, they do not account the impact of disease on people’s mental health and how this can also impact people’s quality of life. As the presented findings suggest, lived experiences highlight a more holistic view of the impact of a disease, rather than a reductionist view of biomedical factors which can be influentill to calculating QALY [8, 9, 29]. Lived experiences could therefore assist policy-makers (and others) across the globe to understand why some people may want to utilise genomic technologies to treat themselves or prevent future generations from being at risk of sharing such experiences. They could also be utilised to educate policy-makers on the reality of living with disease so that they can understand the profound impact austerity measures have on people’s lives.

Some scholars suggest that when assessing the concepts of severity or seriousness, and quality of life, there is a discrepancy in attitudes and perceptions between people living with genetic conditions and those who do not [33, 34]. Other scholars argue that such a discrepancy does not exist [35]. However, this argument is often based on research which explores hypothetical scenarios rather than lived experiences. Thus, while there is a growing range of research which in part or entirety considers the concepts of severity or seriousness as part of their aim [8–10, 29], more research with people who have a range of genomic conditions is needed to ensure a more comprehensive and holistic framework for assessment can be designed.

### Limitations of research

The modest sample size for this research means that findings cannot be conclusive, and further research is needed to verify their validity. Additionally, participants were recruited via online support groups; this method may have biased the sample. And, while the data was critically analysed by the author, others may interpret the data from different perspectives.

## Conclusion

This article argued that valuable insights can be gained from people’s lived experiences and the subjectivities that arise from them in terms of how symptoms of genetic disease are perceived in relation to severity. Findings presented in the article suggest that living with disease often encompasses experiencing a range of symptoms and associations in tandem, and these cumulatively impact a person’s quality of life and mental health. A person’s quality of life was very influential to participant’s perceptions of severity. The article also argued that social, environmental, and economic factors impact a person’s quality of life and their mental health. Thus, future research involving lived experiences with genetic disease could benefit from exploring how frameworks aimed at assessing severity in this genomic age could encompass these factors. A focus on genetic diseases matters because only such diseases would be eligible for, existing or potential, clinical applications of gencomic technologies.

## Data Availability

Access to data generated from and analysed in the research discussed in this article is available by request from the author.

## Appendix 1—adapted excerpts from the interview schedule

**Introduction to Research**

- Brief details of previous research
- Who has been asked to participate and why
- How the research will be used
- Explain what genomic technologies are - inc. which technology this research is focussed on, and distinction between which technologies currently exist for clinical application, and those which could be developed for future clinical applications

**Q 4**. Findings from an online survey I conducted last year revealed that 66% (345/521) of the respondents had a predisposition to be in favour of HHGE to eradicate any disease, regardless of the severity. Should the severity of a disease still be taken into account when considering what conditions to prevent from being inherited, and why?

**Q 5. [Activity]** Following a discussion of what is meant by each symptom or association with disease, ask participants to—rank the following symptoms/associations in order of which they perceive to be most – least impactful considerations on a person’s quality of life. Tell participants that they will be asked to share the reasonings for the rankings at the end of the activity.

- Cognitive degeneration
- Cognitive impairments
- Dependency
- No/poor treatment available
- Pain
- Physical degeneration
- Physical impairments
- Physiological degeneration
- Physiological impairments
- Other
